# Mesoporous starch aerogels production as drug delivery matrices: synthesis optimization, ibuprofen loading, and release property

**DOI:** 10.3906/kim-1912-18

**Published:** 2020-06-01

**Authors:** Akbar MOHAMMADI, Jafarsadegh MOGHADDAS

**Affiliations:** 1 Transport Phenomena Research Center, Faculty of Chemical Engineering, Sahand University of Technology, Tabriz Iran

**Keywords:** Adsorption isotherm, drug release, ibuprofen, optimization, potato starch aerogel, supercritical CO_2_

## Abstract

The aim of this work was to prepare biodegradable starch aerogels as drug carriers. The effective parameters in the synthesis and the optimal values of these parameters were determined using Minitab experimental design software. Ibuprofen was selected as a model drug for the dissolution study and loaded into optimized aerogel during the last solvent exchange step. The Fourier Transform Infrared Spectroscopy (FTIR) analysis showed that ibuprofen has been successfully loaded into the aerogel matrix without any effect on the aerogel nature. The drug loading was calculated to be 29%. The isotherm of ibuprofen adsorption into aerogels matrices followed from the Freundlich isotherm. The in vitro release tests of crystalline ibuprofen and ibuprofen-loaded potato starch aerogel were investigated with simulated gastric and intestinal fluids in USP 2 apparatus. It was shown that the dissolution rate of ibuprofen could be dramatically changed. Also, an improvement in the dissolution rate of ibuprofen was achieved by performing the dissolution test first in the gastric medium for 120 min and then in the intestinal medium for up to 270 min. A higher release rate (100%) was observed at the end of the in vitro experiment.

## 1. Introduction

Aerogels are the lightest porous solid nanomaterials produced by the 2-stages sol-gel method through substitution of liquid in gel pores with air [1,2]. Since the preparation of the first aerogels by Kistler in 1931, these substances have found numerous applications in thermal insulation [3–5], catalyst supports [6,7], space engineering [4], humidity sensors [8], wastewater treatment [1,4], nuclear fusion reactors [6], electrodes [9], electrical circuits (due to their low dielectric constant) [8], solar energy collectors [4], super high-density capacitors [3,4], adsorbent (due to their high porosity and surface area) [9], and carriers for delivery systems [10–12]. About 22% of biomedical applications of aerogels are related to drug delivery [13]. Biocompatibility and biodegradability are highly necessary in drug delivery applications due to their direct relationship with human health. Silica aerogels possess interesting properties including biocompatibility, but they are not biodegradable and may be harmful for in vivo applications. For this reason, their use in biomedical applications has been limited [11,14,15]. Recently, polysaccharide-based aerogels have been synthesized and scaled-up (from bench scale to pilot scale) for drug delivery applications [16]. Polysaccharides, as a class of biopolymers, are composed of single or more simple sugar monomers. Due to their availability, recyclability, nontoxicity, good biological performance, and enzymatic degradation they have found applications in pharmaceuticals, drug carriers, and tissue engineering [17–20]. Polysaccharides can convert to hydrogel at a supersaturated concentration in the presence of physical or chemical cross-linkers owing to their hydrophilic structure [19]. Conventional polysaccharides such as alginate, pectin, chitin, chitosan, starch, marine polysaccharides, hyaluronic acid, cellulose derivatives (like hydroxyethyl cellulose and carboxymethyl cellulose) have been used in drug delivery systems [18,19]. Polysaccharide aerogels possess outstanding features of inorganic aerogels such as porous structure [13,14,15,19] and can be used as drug carriers with high potential and without limitations. In a study by García-González et al., starch aerogels were loaded with ketoprofen and benzoic acid and specific loading values of 1.0 × 10^-1^ and 1.7 × 10^-3^ g/m^2^ were achieved for ketoprofen and benzoic acid, respectively [17]. Mehling et al. (2009) synthesized polysaccharide aerogel from starch and alginate materials and showed that polysaccharide aerogels have a high potential as drug carriers [14]. Gonçalves et al. (2016) synthesized alginate aerogel and alginate-pectin and alginate-carrageenan hybrid aerogel with a specific surface area higher than 300 m^2^/g through an emulsion-gelation method combined with drying by supercritical carbon dioxide [10]. Chang et al. (2010) synthesized starch-derived carbon aerogels by ambient pressure drying method [21]. In pharmacokinetic topics, drug dissolution is the requisite for drug absorption in the body to attain the subsequent drug response, particularly for orally administrated drugs. Nevertheless, in accordance with the Biopharmaceutics Classification System (BCS), about 70% of drugs belong to class II drugs which show low solubility and high permeability [22]. When these types of drugs are administered in crystalline form, they get out of the gastrointestinal tract before complete dissolution and absorption into the blood circulation system. The therapeutic concentration range of a drug in the blood can be achieved by dose augmentation. Studies have shown that solubility and bioavailability of poorly water-soluble drugs can be increased by loading into porous matrices as molecular dispersions. Incorporation of drugs into porous matrices during the solvent exchange step is one of the simple and low-cost methods of drug loading [23]. Ibuprofen, a nonsteroidal antiinflammatory drug, is practically insoluble in water and soluble in ethanol. Therefore, it can be loaded into porous matrices during the solvent exchange step in order to improve its solubility and bioavailability. In the synthesis of organic and inorganic aerogels, researchers have used the classic optimization method of OFAT (1-factor at a time). In this method, effective parameters in synthesis are either not investigated or they are studied case by case, where the interaction between the parameters is not considered. Moreover, none of the researchers have optimized the synthesis process and obtained the optimal point of synthesis. The aims of this work are to optimize the synthesis of potato starch aerogels, to load optimized aerogels with ibuprofen in order to improve its solubility and bioavailability, to characterize the drug-loaded and unloaded aerogels, to study the adsorption isotherm, and to determine the loading capacity of ibuprofen. For this purpose, the effect of 2-ways and 3-ways interactions between the parameters and the effect of the main parameters (main effects) on the synthesis of starch aerogels were studied using Minitab 17.3.1 experiment design software by central composite design method. In addition, the optimal value of effective parameters was obtained. Ibuprofen, a class II drug, was selected as a model drug and loaded into the optimized tablet-shaped potato starch aerogel by adsorption from the ibuprofen/ethanol solution during the last step of the solvent exchange process. Adsorption isotherm and the loading capacity of ibuprofen were determined. Finally, in vitro release tests of ibuprofen were carried out using a USP 2 apparatus in both simulated gastric (pH 1.2) and intestinal (pH 7.4) fluids.

## 2. Materials and methods

### 2.1. Materials

Potato starch with a density of 1.05 g/cm^3^ was supplied from Samchun pure chemical company (South Korea). The applied deionized water was produced in the lab. Ethanol with 99.9% purity was purchased from Merck company. Ibuprofen was from Sigma Aldrich.

### 2.2. Starch aerogel preparation

Based on the design of the experiment, a specific amount of starch was mixed with deionized water. The mixture was stirred and heated until a viscose milky solution was obtained. The obtained gel was poured into tablet-shaped polyethylene molds with a diameter of 16 mm and a depth of 10 mm. The molded gels were kept at 4 °C or room temperature for retrogradation, as proposed by the design of the experiment software. To protect the porous structure of the gel and prevent its shrinkage during drying, the water content of the gel pores must be replaced by a fluid with low surface tension and high vapor pressure [24]. The water content of the gel will shrink the gel and elongate the drying process due to its high surface tension and low vapor pressure [25]. To exchange the water in the gel pores with ethanol, aged hydrogels were immersed in 40%, 70%, and 90% (v/v) ethanol and pure ethanol (twice) for t_*S.E*_ (time period of solvent exchange) [14]. In the next step, tablet-shaped aerogels were obtained by extraction of ethanol with SC–CO_2_ in an autoclave for 4 h. The details of supercritical drying were described elsewhere [26]. Supercritical drying conditions were as follows: 10 < p < 15 MPa, 40 < T < 50 °C, CO_2_ flow rate 1 L/min. By depressurizing the system at CO_2_ flow rate of 1 L/min and temperature of 40 °C, supercritical CO_2_ was first converted to gaseous form and then replaced by air when the autoclave reached ambient conditions.

### 2.3. Design of experiment

As mentioned in the introduction, Minitab 17.3.1 experiment design software was applied in order to investigate the effect of 2-way and 3-way interactions between the parameters, the effect of main parameters (main effects) on the synthesis of starch aerogels, and to optimize the process parameters such as weight percentage of starch in sol (x), retrogradation time (t_R_), time period of solvent exchange (t_S.E_), and retrogradation temperature (T_R_). A response surface design is a set of advanced techniques of design of experiments (DOE) that optimize responses. This methodology is often used to refine models after determining important factors; especially, when a curvature is suspected in the response surface. A central composite design is the most commonly used response surface designed experiment [27]. Therefore, this method was applied to design and optimize the experiments. Here, important factors and their levels were selected according to previous studies. The weight percentage of starch in sol (x), retrogradation time (t_R_), and time period of solvent exchange (t_S.E_) were considered as the main factors, while retrogradation temperature (T_R_) was regarded as a block. The lower levels of x, t_R_, and t_S.E_ were 6%, 3 days, and 4 h, respectively. The upper levels of x, t_R_, and t_S.E_ were 14%, 5 days, and 20 h, respectively. Block 1 and block 2 were considered for T_R_ = 4 ◦C and T_R_ = 25 ◦, respectively. Table 1 provides the experiments proposed by Minitab software.

**Table 1:** Experiments predicted by Minitab experimental design.

t_R_(day>	w/w% Starch (x)	t_S.E_ (h)	Blocks (T_R_)		PtType	RunOrder	StdOrder
**4**	10	12	1	*T_R_* =4◦	0	1	12
**4**	10	12	1			0	2	9
**5**	14	4	1			1	3	7
**3**	14	4	1			1	4	3
**3**	6	4	1			1	5	1
**5**	6	20	1			1	6	6
**3**	6	20	1			1	7	2
**4**	10	12	1			0	8	10
**5**	14	20	1			1	9	8
**5**	6	4	1			1	10	5
**4**	10	12	1			0	11	11
**3**	14	20	1			1	12	4
**4**	16	12	2	*T_R_* =25◦	--1	13	16
**4**	10	24	2			-1	14	14
**4**	10	12	2			0	15	20
**2.5**	10	12	2			-1	16	17
**4**	10	0	2			-1	17	13
**5.5**	10	12	2			-1	18	18
**4**	10	12	2			0	19	19
**4**	4	12	2			-1	20	15

### 2.4. Drug loading, drug content determination, and in vitro release experiments

Ibuprofen (Sigma Aldrich) was loaded into the tablet-shaped potato starch aerogel during the last solvent exchange step. This approach is regarded as the simplest approach and an adaptable method to load poorly water-soluble drugs [23]. In order to determine the maximum drug loading capacity, the tablet-shaped alcogels were immersed in ibuprofen/ethanol solution having different ibuprofen concentrations (10, 20, 30, 40, 50, 60, 70, 80, 90, 100, and 110 mg/mL) for 24 h (the optimum time period of solvent exchange) in a closed batch system to prevent evaporation of the liquid. Then ibuprofen-loaded alcogels were dried with supercritical CO_2_ in order to obtain drug-loaded aerogels. In order to determine the drug content in aerogels, the drug-loaded aerogels were pounded and mixed with 40 mL of ethanol. After 3 h of stirring at 300 rpm (round per minute) and 37 ±0.1 °C and sonicating for 30 min, the solution was filtered through a Teflon 0.45 μm sieve and the absorbance was measured spectrophotometrically using a Shimadzu UV-3150PC UV/Vis spectrophotometer [14,17]. Then, the concentration was calculated using the calibration curve ibuprofen-ethanol. Drug dissolution tests of ibuprofen-loaded tablets and crystalline drugs were conducted in triplicate using the USP 2 paddle apparatus with a constant agitation speed of 50 rpm at 37 ±0.1 °C [28]. The in vitro release tests were carried out in simulated intestinal fluid (SIF, phosphate buffer solution, pH 7.4), and simulated gastric fluid (SGF, HCl aqueous solution, pH 1.2). Also, in order to simulate the behavior of the tablets after oral administration, the simulated gastric medium (pH 1.2) was replaced by a simulated intestinal medium (pH 7.4) after 120 min. At determined time intervals, aliquots (2 mL) of dissolution media were sampled from the dissolution vessel and the same amount of fresh dissolution medium was added to maintain constant volume within the vessel. The ibuprofen concentration was determined by means of a Shimadzu UV-3150PC UV/Vis spectrophotometer at a wavelength of 265 nm.

### 2.5. Characterization of the synthesized samples

The apparent density of the aerogel was measured by mass to volume ratio (ρ = mv^-1^) of the aerogel, in which mass was measured by the microbalance with the accuracy of 10^-4^ g and the volume of aerogel was measured by filling aerogel in the measuring cylinder of a known volume. The residual moisture content of the starch aerogels was determined by heating the aerogels at 110◦ for 24 h and measuring the weight loss of the samples by a smart scale with the accuracy of 10^-4^ g [14]. The structural properties of the aerogels were determined by the standard nitrogen gas adsorption method using a surface area analyzer (ASAP 2020). Nitrogen adsorptiondesorption isotherms were obtained at 77 K. Prior to analysis, samples were heated at 110◦ for 2 h in a vacuum oven [14]. Specific surface area and pore size distribution were calculated by Brunauer-Emmett-Teller (BET) and Barrett-Joyner-Halenda (BJH) methods. The volume of pores and particle size were also determined by BET analysis. For the qualitative study of functional groups and chemical bands in the structure of starch aerogels and potato starch precursor, FTIR analysis (BRUKER-TENSOR 27) was applied. The morphology and microstructure of drug-loaded and unloaded starch aerogels were studied using field emission scanning electron microscope (FESEM) (TESCAN-MIRA III).

## 3. Results and discussion

### 3.1. Temperature pattern for hydrogel preparation

**Figure 1:**
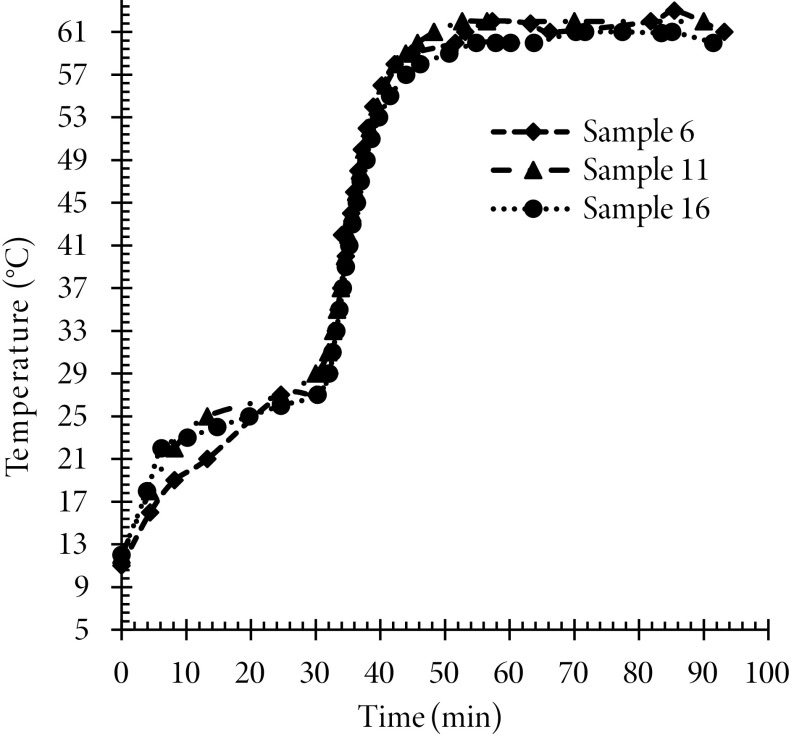
Temperature pattern used in the preparation of starch hydrogel.

Gel preparation is one of the vital stages in the sol-gel process as the characteristics of synthesized aerogels highly depend on the gelation step. Potato starch sol can be converted into hydrogel at concentrations greater than the saturated concentration in the presence of temperature physical cross-linker. Therefore, the applied temperature procedure is very important in the sol conversion into hydrogel. The indicators of conversion of sol into gel are the change of sol color into an opalescent solution and a sudden rise of its viscosity. In the present study, this critical point occurred at 62 ◦ . Figure 1 shows the temperature pattern applied for potato starch sol in order to prepare the hydrogel. The potato starch-water solutions, with weight percentages proposed by the design of the experiment, were first stirred for 30 min with low-temperature variations. This time was enough to prepare potato starch sol; at this stage, the starch hydrophilic granules were swollen by water absorption and dissolved by heating gradually. Amylose molecules were extracted from the potato starch structure by the dissolution of swollen granules, therein resulting in irreversible physical changes and degradation of the granular structure. By gradual reheating, the ratio of residual granular structure in the sol would be reduced, and consequently, amylose would be released from the starch structure and sol crystallization would be increased. When the sol temperature reached 62 °C, the solution became opalescent and its viscosity increased suddenly. The occurrence of these physical changes demonstrated the beginning of gelation and conversion of sol into hydrogel. However, these changes did not imply completion of the gelation process because the time of the beginning of gelation was not high enough to provide complete dissolution of all swollen starch granules inside the solution and to the optimal increase of viscosity to form an acceptable aerogel structure. Therefore, the resulting solution was stirred at an agitation rate of 400 rpm (at 62 °C) until proper viscosity was obtained. Our experiments showed that with increasing temperature of gelation, the gel network became denser and its structure became stronger and more rigid. In this case, the drying of alcogels resulted in low porosity aerogels.

#### 3.2. Properties of potato starch aerogels

##### 3.2.1. Equilibrium moisture content of aerogels

**Figure 2:**
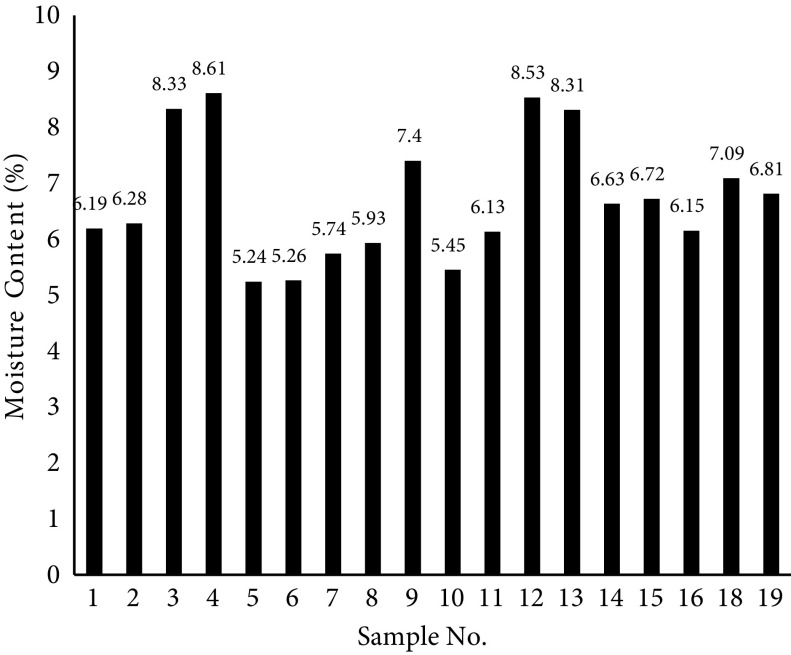
Equilibrium moisture content of synthesized samples.

The total amount of liquid in the alcogel is called total moisture content. Some of this moisture that is easily removed by the drying method is known as free moisture content. However, the moisture content that is removed by heating at high temperatures is the equilibrium moisture content [29]. During the drying process of alcogels, all moisture (water and ethanol) in the gel pores may not be removed from the gel structure; therefore, some moisture may still remain inside the aerogel pores. To measure the equilibrium moisture, a certain amount of starch aerogel was placed into a vacuum oven at 110 °C for 24 h. The weight loss of the aerogels after 24 h of heating indicated the residual moisture content. Figure 2 shows the equilibrium moisture content of the samples synthesized according to the conditions proposed by the design of the experiment. The average moisture content of the synthesized samples is 6.77% of the moist aerogel. According to this Figure, the moisture content of the samples depends on the weight percentage of potato starch in the aerogel, so that the greater the weight percentage, the higher the moisture content. A reason for this is the formation of weak hydrogen bonds between potato starch molecules and those of water; therefore, the greater the number of starch molecules in aerogel structure, the more bounded water there will be and moisture content is more likely to increase.

##### 3.2.2. Microstructure of aerogels

**Figure 3:**
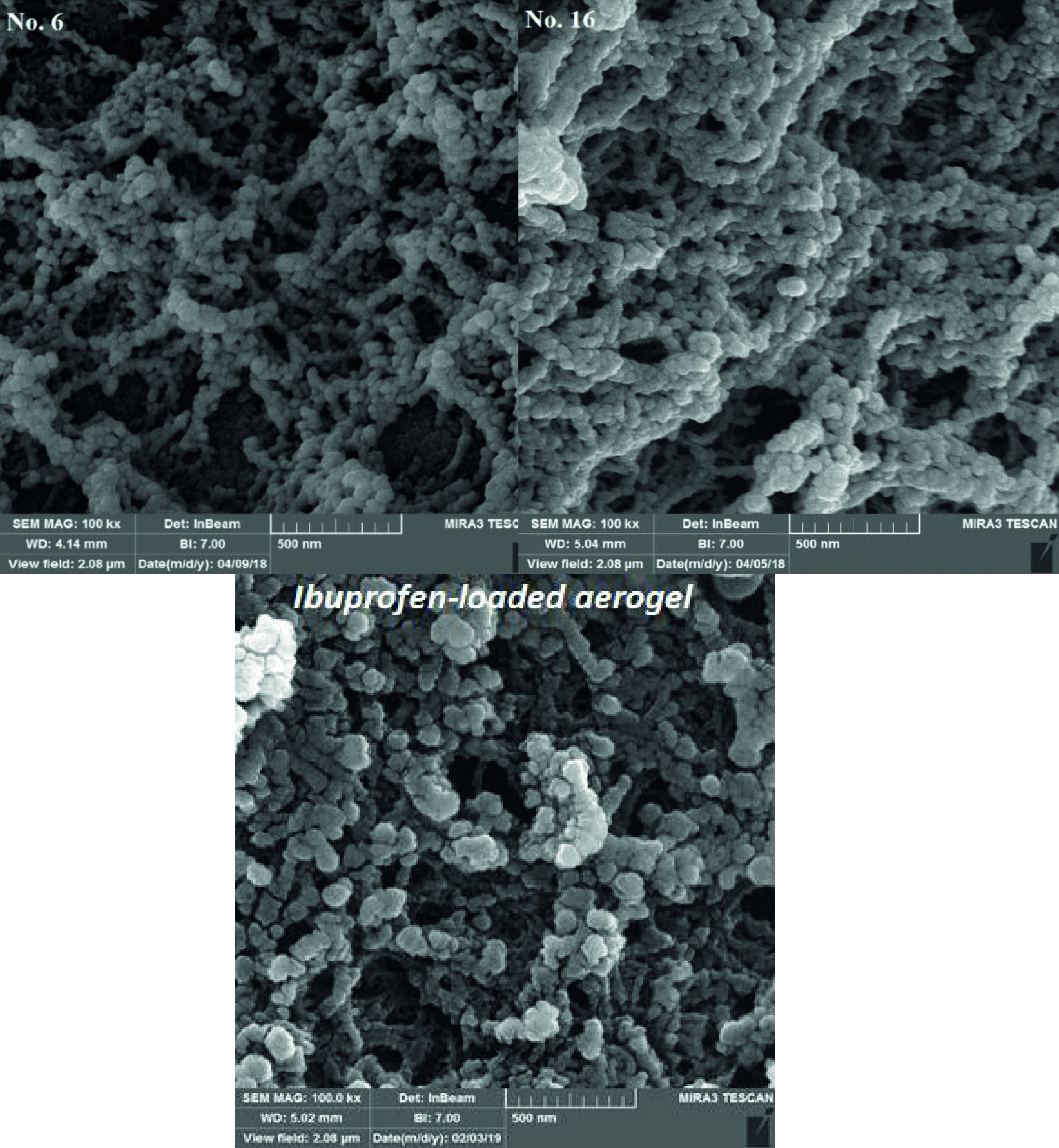
FESEM images of pure potato starch aerogel and ibuprofen-loaded potato starch aerogel synthesized at the optimal point.

To investigate the morphology and microstructure of the synthesized samples, 2 samples were randomly selected from blocks 1 (sample 6) and 2 (sample 16) for FESEM imaging at the nanoscale. FESEM pictures of samples (Figure 3) clearly show a porous network structure containing spherical solid clusters. Also, these pictures exhibit uniform particle distribution that contains a mean particle size of about 100 nm. According to FESEM images, the synthesized samples possessed an open pore structure that became more complete with the reduction of starch percentage as well as retrogradation temperature. In the synthesis of aerogels, the network structure, rather than the granular structure, should be extended until aerogels with high surface area are produced. By extension of the 3-dimensional network, the mean size of the pores decreased resulting in an increase of pore volume, porosity, and specific area. Temperature pattern during gelation and retrogradation temperature can affect this process so that with decreasing retrogradation temperature, nucleation increases and crystallization rate decreases. Therefore, retrogradation at a lower temperature can increase the number of small crystals, thereby enhancing the surface area of aerogels. This trend can be seen for potato starch aerogels with a retrogradation temperature of 25 °C and 4 °C. BET results also confirmed this trend. The FESEM micrograph shows that retrogradation at low temperatures forms a regular network structure of starch microparticles. These microparticles grow in the form of branching filaments and produce a mesoporous aerogel matrix. By increasing retrogradation temperature, the crystallization rate increases and microparticles agglomerate to form bigger aggregates, linked primary microparticles, thereby causing the aerogels with lower porosity to produce. Also, a FESEM image of ibuprofen-loaded aerogel synthesized at the optimal point is shown in Figure 3. This Figure reveals no considerable morphological variations in the aerogel structure due to ibuprofen loading via LSES method (loading at the solvent exchange step). It is evident that the loading of ibuprofen does not affect the nanoporous structure of the aerogels and no ibuprofen crystals are observed on the surface of the particles or separately along with the particles.

##### 3.2.3. Structural properties of aerogels

Specific surface area and porosity characteristics of the synthesized samples (in accordance with the condition proposed by the design of the experiment) are listed in Table 2. These values meet the characteristics of the potato starch aerogel (S_BET_ = 72.5 ±10.5 m^2^/g, bulk density = 0.46 ±0.06 g/cm^3^, overall pore volume = 0.47 ±0.07 cm^3^/g, average pore radius = 7.2 ±0.01 nm) prepared by Mehling et al. [14]. Regarding Table 2, the samples synthesized at T_R_= 4◦ possess a greater surface area than those synthesized at T_R_= 25◦. With a decrease of T_R_, the nucleation rate increases, and the crystallization rate decreases. Hence, the number of small crystals increases resulting in enhanced surface area. The influence of other effective parameters on the synthesis is presented in the design of the experiment analysis. The average pore diameter of the samples is in the range of 12.2 to 29.1 nm (mesopore). The average pore diameter data shows that T_R_ has a significant effect on the formation of the gel network during the aging process (retrogradation) and on the shrinkage of aerogels during the drying process. Therefore, retrogradation in lower temperatures results in aerogels with lower shrinkage and open-pore grid structure (Figure 3) with a pore size of 12.2–17.3 nm. While the aerogels synthesized at T_R_= 25 ◦ contain a denser network compared to the aerogels in block 1 (T_R_ = 4◦) with a pore size of 18.9–29.1 nm. Specific surface area of samples synthesized at T_R_= 4◦ is more significant than that of the samples synthesized at T_R_= 25◦. Specific surface area depends on density and pore size, which has a reverse relationship with density and pore size [30]. Therefore, it is logical that samples synthesized at T_R_= 4◦ have lower density and average pore diameter. To interpret the effect of synthesis parameters on the properties of the synthesized aerogels, 2-way and 3-way interactions between the parameters should be investigated. This is possible by statistical analysis of data addressed in the design of the experiment section.

**Table 2:** 

Density (g/cm^3^) ±0.05	Average pore width (nm) ±0.01	Overall pore volume (cm^3^/g) ±0.06	BET surface area (m^2^g) ±10	Sample No.
0.37	14.2	0.46	65	1
0.38	14.1	0.47	66	2
0.43	16.1	0.33	51	3
0.46	16.4	0.32	50	4
0.49	17.3	0.35	49	5
0.26	13.4	0.40	67	6
0.35	15.4	0.39	62	7
0.32	13.5	0.49	69	8
0.20	12.2	0.42	70	9
0.45	16.5	0.35	50	10
0.35	13.1	0.49	63	11
0.29	12.9	0.34	68	12
0.35	20.2	0.33	50	13
0.32	18.9	0.38	49	14
0.56	28.7	0.31	42	15
0.46	23.5	0.32	45	16
aerogel was not synthesized				17
0.50	25.4	0.25	47	18
0.56	29.1	0.26	46	19
aerogel was not synthesized				20
0.39	13.7	0.45	64	Optimal sample

##### 3.2.4. FTIR analysis

**Figure 4:**
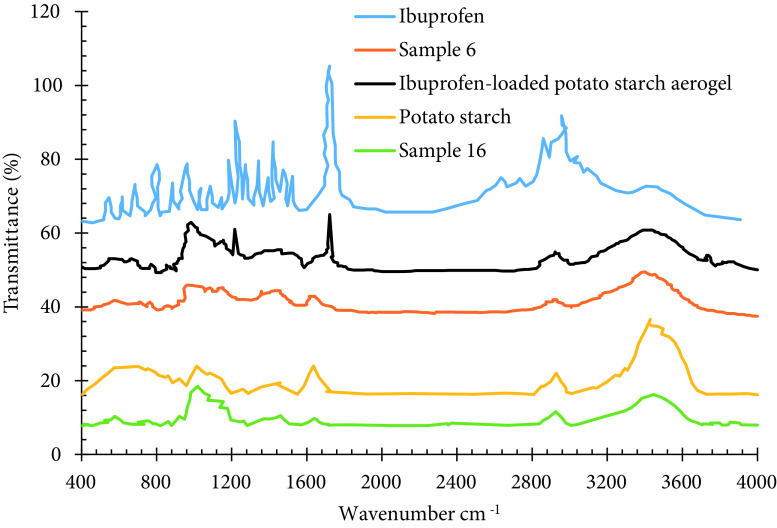
FTIR spectra of potato starch, ibuprofen, ibuprofen-loaded, and unloaded potato starch aerogel.

To identify different functional groups in the structure of ibuprofen-loaded and unloaded potato starch aerogel, potato starch (precursor), pure ibuprofen, and to confirm the loading of ibuprofen in the aerogel matrix, Fouriertransform infrared spectroscopy (FTIR) was carried out as shown in Figure 4. In the drug-loaded and unloaded potato starch aerogel and potato starch, the absorbance peaks of 1018 cm^-1^ represent the anhydroglucose C–O–C stretching vibration. The characteristic peaks at around 1080 cm^-1^ and 1158 cm^-1^ observed at potato starch aerogels can be ascribed to the C–O–H bond. The absorption peak at around 1460 cm^-1^ corresponds to the twisting vibration of −CH_2_ group. The principal band between 1500–2000 cm^-1^ is due to C=C and C=O. The intensity of the C=C and C=O peaks in the structure of potato starch is much larger than that of potato starch aerogels. During the transformation of starch powders into hydrogels, despite the unchanged nature of starch powder, a series of irreversible physical changes and granular structure degradation occur. After that, gel structure reorganized and partial crystallization occurred during the cooling and retrogradation process. Therefore, the intensity of the structural C=C and C=O peaks of potato starch was higher than that of potato starch aerogel. According to FTIR analysis, the intensity of O–H peak of potato starch is higher than that of potato starch aerogel. This may be attributed to the higher moisture content of powdered potato starch, as the synthesized samples were degassed under vacuum for 2 h at 110 °C before FTIR analysis. The FTIR spectrum of ibuprofen shows the absorption bands at 1722 cm^-1^ and 2924 cm^-1^, corresponding to C=O and C–O stretching. The band at 1231 cm^-1^ was assigned to C–O stretching. Other various characteristic peaks were observed at 1456 cm^-1^ for ring vibration, at 2959 and 2864 cm^-1^ for CH stretching. The ibuprofen-loaded potato starch aerogel exhibited both the features of pure ibuprofen and potato starch aerogel. This similarity between the spectra confirms the successful loading of ibuprofen and indicates that ibuprofen does not react with potato starch aerogel.

#### 3.3. Data analysis of experimental design

The aim of this work is to optimize the synthesis of potato starch aerogel for drug loading applications. Since specific surface area plays the most important role in drug loading, it was selected as the response in the optimization procedure and the effect of the synthesis parameters on this feature was addressed. The response surface regression results presented in Table 3 are provided as the analysis of variance. The P-values of this table determine the effective parameters on the specific surface area (response). Usually, the significance level of 0.05 works well. For effective parameters, the P-value is less than 0.05, while ineffective parameters possess P-values greater than 0.05 [27]. Regarding the P-value of the model (0.000 < 0.05) and P-value of the lack-of-fit (0.097 >0.05), it was determined that the model is acceptable and desirable for statistical analysis of the data. The analysis of variance table (Table 3) shows that t_S.E_, x, block 1 (TR= 4 C), and the square of main effects (x*x and t_S.E_ ∗ t_S.E_) were effective on the result of experiments (specific surface area), whereas t_R_, t _R_ ∗ t_R_, and the interactions between the parameters were ineffective. It should be noted that the effect of t_R_, t_R_ ∗ t_R_ , and 2-way interactions cannot be neglected, and it is better to say that their effect was less than that of x, T_R_ , t_S.E_, x*x, and t_S.E_ ∗ t_S.E_. Table 3 lists the type of effect of parameters on the response (reverse or direct effect) and their coefficients to obtain the regression equation of the response. According to this table, t_S.E_ (time period of solvent exchange) had the highest direct effect on the response (leading to an increase in the specific surface area). The square of the main effects exhibited an inverse effect on the response. This means that the relationship between the parameter and the response follows a curved line. The proposed regression equation by software, which is the averaged equation on both blocks, is presented by Eq. (1).

(1)BETsurfacearea=2.4+1.739tS.E++2.49x+9.70tR--0.0483tS.E*tS.E--0.0960x*x--1.092tR*tR+0.0273tS.E*x+0.0781tS.E*tR--0.094x*tR

**Table 3:** Analysis of variance results.

P-value	F-value	Adj MS	Adj SS	DF	Coef	Source
0.000					56.224	Constant
0.000	54.99	243.98	2439.82	10		Model
0.000	273.34	1212.74	1212.74	1	8.006	Blocks 1
0.000	86.50	383.79	1151.38	3		Linear
0.000	244.73	1085.78	1085.78	1	9.320	Time period_S.E. (h)
0.007	12.19	54.08	54.08	1	2.080	wt% starch
0.142	2.60	11.52	11.52	1	0.960	Retrogradation time (day)
0.003	10.31	45.74	137.23	3		Square
0.001	22.11	98.07	98.07	1	-3.092	Time period_S.E. (h) * Time period_S.E. (h)
0.044	5.46	24.22	24.22	1	-1.537	wt% starch * wt% starch
0.131	2.76	12.24	12.24	1	-1.092	Retrogradation time (day) * Retrogradation time (day)
0.535	0.78	3.46	10.38	3		2-way interaction
0.270	1.38	6.12	6.12	1	0.875	Time period_S.E. (h) * wt% starch
0.423	0.70	3.12	3.12	1	0.625	Time period_S.E. (h) * Retrogradation time (day)
0.627	0.25	1.13	1.13	1	-0.375	wt% starch * Retrogradation time (day)
		4.44	39.93	9		Error
0.072	5.01	6.89	34.43	5		lack-of-fit
		1.38	5.50	4		Pure error
			2479.75	19		Total

**Figure 5:**
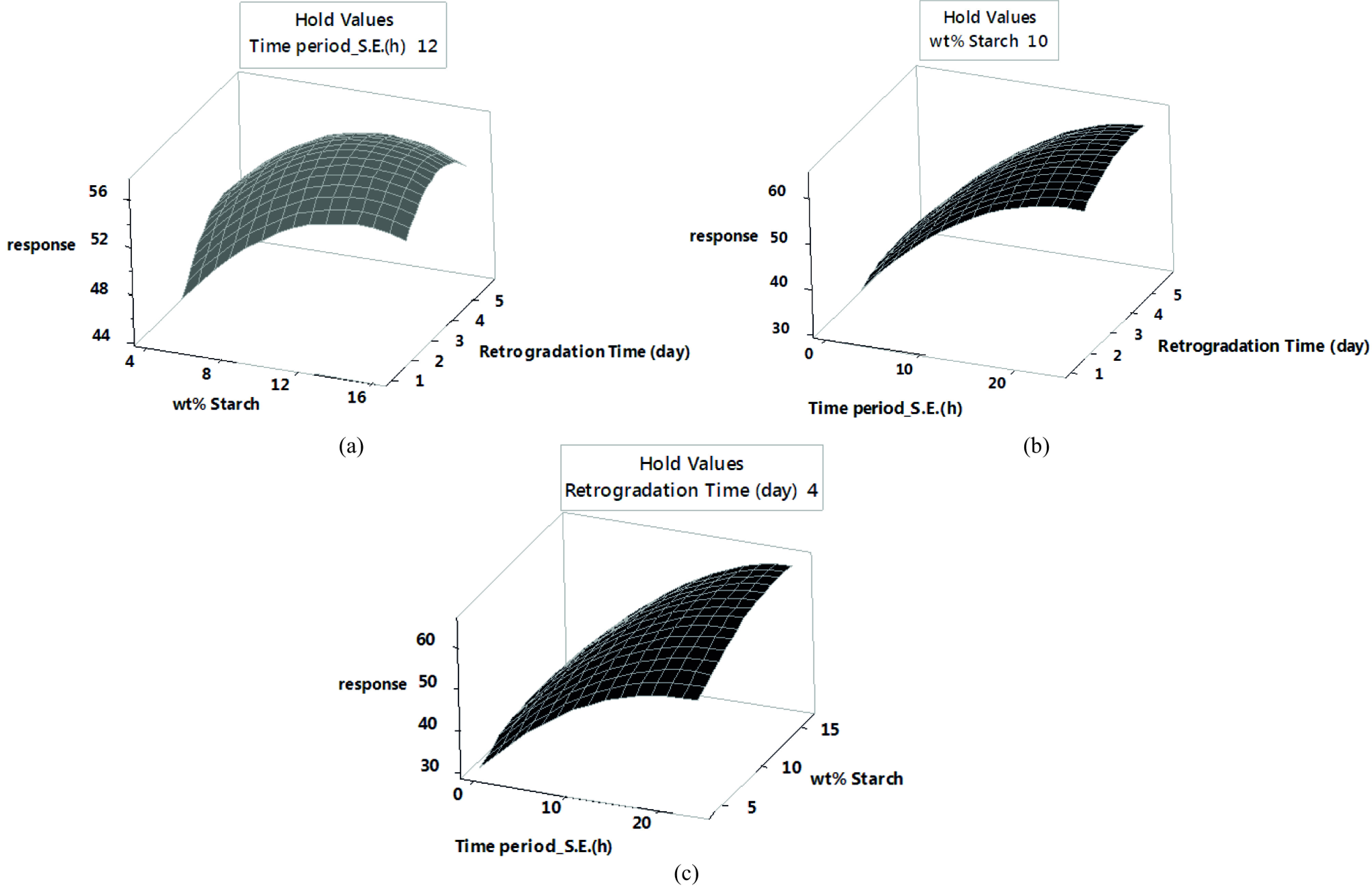
Surface plot of BET surface area vs. synthesis parameters.

To better understand the effect of synthesis parameters on the response (specific surface area), a 3-D surface plot of BET surface area was plotted versus the synthesis parameters in Figure 5. Figure 5a shows that by keeping t_S.E_ constant at the central point (12 h), the specific surface area first increased with the increment of x and t_R_ and then decreased. Thus, this diagram contains an extremum point, with the global maximum of the surface area approximately at the point near the upper level of t_R_ and x = 10%. The surface area is plotted against t_S.E_ and t_R_ in Figure 5b, while x is fixed at its central point, i.e. 10%. According to this diagram, the slope of the graph is steeper than that of the previous one and its maximum point is approximately at the point near the upper level of tS.E and t_R_. The trend of surface area variations relative to x and t_R_ is depicted in Figure 5c. In this diagram, the third parameter (t_R_) is kept constant at its midpoint, i.e. 4 days. The trend of variation of the specific surface area in this diagram is almost the same as Figure 5b.

### 3.4. Response optimization

**Figure 6:**
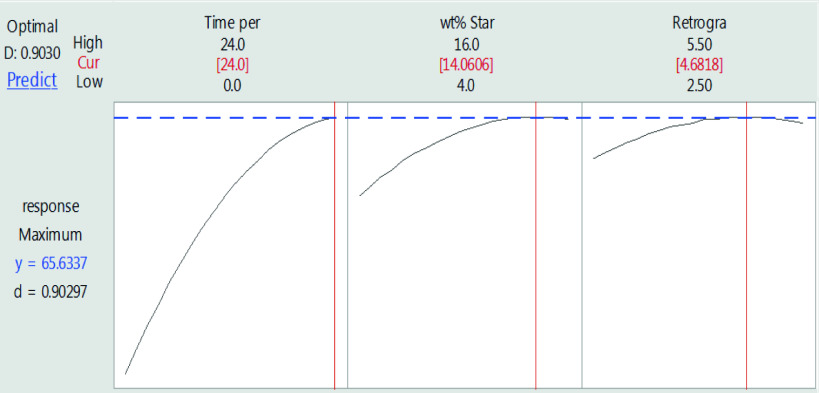
Response[de Sá, 2017 #190] optimization plot.

In the present study, to investigate the effect of the mentioned parameters, their interactions, and square of main effects on the specific surface area, the design of experiment method and the central composite design model were used. The major objective was not to reduce the number of experiments but to obtain the best acceptable response considering the limitations and requirements of the problem. Therefore, the optimal parameters were obtained to achieve the best response with the least cost and time. The optimization diagram along with the optimal parameters is provided in Figure 6. Since the target function of optimization was to maximize the response, and since block 1 directly affected the response, optimal experiments should be conducted on block 1. Figure 6 shows that the optimal values of t_S.E_, x, and t_R_ are 24 h, 14%, and 4.7 days, respectively. Response optimization predicted the optimal specific surface area of 65.6 m^2^/g. To validate the optimization result, a sample was synthesized with optimum parameters in block 1 and characterized. The structural properties of the optimized sample are presented in Table 2. Since the optimized sample is structurally similar to the samples synthesized in accordance with the design of experiments (samples 6 and 16), descriptions related to the analysis of results of the previous section hold for the optimal sample as well.

### 3.5. Ibuprofen adsorption

**Figure 7:**
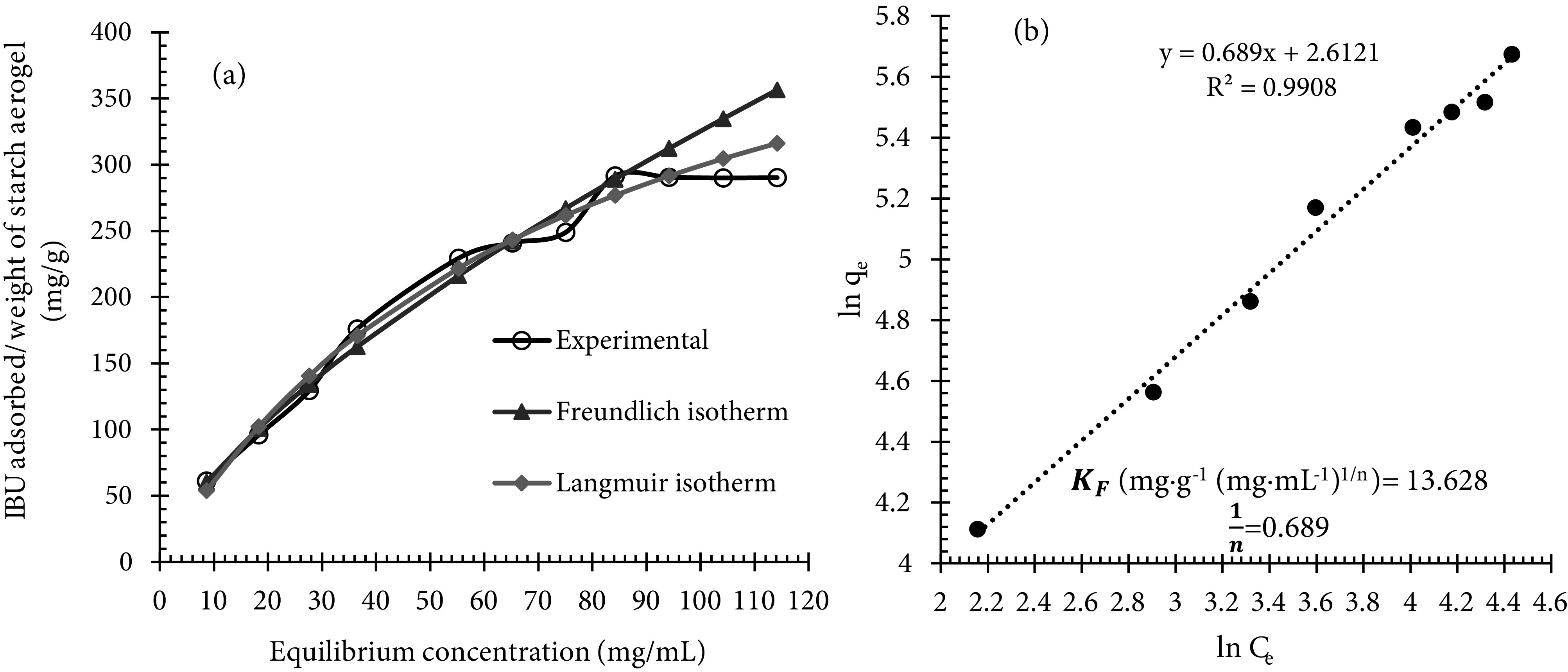
Ibuprofen adsorption isotherm onto the potato starch aerogel (a) and Freundlich isotherm plot of ibuprofen adsorption onto the potato starch aerogel (b).

The optimized potato starch aerogel was loaded with ibuprofen during the last solvent exchange step. Figure 7a shows ibuprofen adsorption isotherm for the optimized potato starch aerogels. This Figure shows that the maximum equilibrium adsorption capacity and specific loading of ibuprofen are approximately 290 mg/g (29 wt% drug loading) and 4.5 ×10^-3^ g/m^2^, respectively. These values meet with the specific loading of some poorly water-soluble drugs listed in Table 4. This table shows that specific loading of poorly water-soluble drugs is in the order of 10^-3^ g/m^2^. Ibuprofen (model drug in this work) belongs to class II drugs and its specific loading was also obtained in the order of 10^-3^ g/m^2^. Regarding Table 4, the specific loading of ibuprofen obtained in this work is in good agreement with the results obtained by other researchers. This result can be explained by the fact that smaller pores enhance capillary forces. This increase causes capillary condensation and thus increment drug loading. Therefore, by loading poorly water-soluble drugs into potato starch aerogel, the solubility and bioavailability of these types of drugs could be improved. Because ibuprofen was adsorbed on a molecular level, the Langmuir and Freundlich isotherm models [34,35] were selected to evaluate the adsorption of ibuprofen in potato starch aerogel. The linear forms of Langmuir and Freundlich equations are represented by Eqs. (2) and (3), respectively.

**Table 4:** 

Drugs	Matrix	Specific loading (g/m^2^)	Reference
Ibuprofen	Potato starch	1-2x 10^-3^	[14]
	Eurylon 7 starch	2-4x 10^-3^	
	Alginate	0.7-1.1x 10^-3^	
	Silica	0.42x 10^-3^	[31]
	Starch	1.4x 10^-3^	
	Alginate	1x 10^-3^	
	Silica-gelatin	0.34x 10^-3^		[32]
Artemisinin	Silica	0.35x 10^-3^	[31]
	Starch	0.18x 10^-3^	
	Alginate	0.30x 10^-3^		
Ketoprofen	Silica-gelatin	0.20x 10^-3^	[32]
	Alginate	0.67x 10^-3^	[10]
	Alginate-pectin	0.49x 10^-3^	
	Alginate-carrageenan	0.41x 10^-3^	
	Silica	0.15x 10^-3^	[17]
	Alginate	0.23x 10^-3^	
	Pectin	0.35x 10^-3^	
	Starch	0.10x 10^-3^		
Triflusal	Silica-gelatin	0.41x 10^-3^	[32]
Quercetin	Alginate	0.10x 10^-3^	[10]
	Alginate-pectin	0.14x 10^-3^	
	Alginate-carrageenan	0.10x 10^-3^		
Nifedipine	High-methoxyl pectin	0.57x 10^-3^	[33]

(2)Ceqe=1qmKL+Ceqm

(3)lnqe=lnKF+(1n)lnCe

Where q_e_ (mg/g) is the equilibrium adsorption capacity; C_e_ (mg/mL) is the equilibrium concentration of the adsorbate; q_m_ (mg/g) is the maximum adsorption capacity; K_L_ (mL/mg) is the Langmuir constant, K_F_ (mg/g (mg/mL)^1/n^) and n are Freundlich constant and heterogeneity factor, respectively. Figure 7b shows the linear plots of Freundlich isotherm for ibuprofen adsorption onto potato starch aerogels. The parameters of isotherms (q_m_, K_L_, K_F_, and n) can be calculated from the gradient and intercept to characterize the adsorption process. The results indicate that ibuprofen adsorption isotherm is well-fitted with the Freundlich isotherm model, indicating that the ibuprofen adsorption process is related to multilayer adsorption on the heterogeneous surface [36]. A variation in the gradient of Freundlich isotherm (1/n) (1/n related to the adsorption intensity or surface uniformity) between 0 and 1 is associated with a chemisorption process, which is more heterogeneous as the value gets closer to 0 [34,35].

### 3.6. Drug release experiments

**Figure 8:**
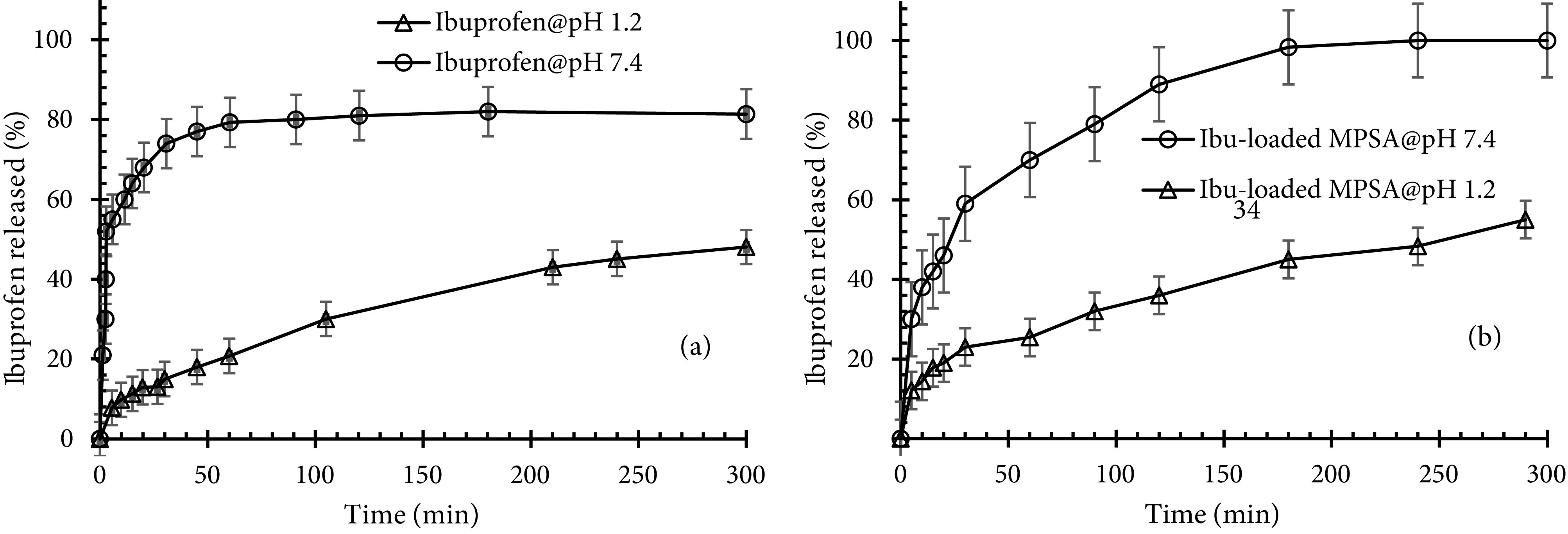
Release kinetics of crystalline ibuprofen within SGF and SIF (a) and ibuprofen release from potato starch aerogel in SGF and SIF (b) (MPSA: mesoporous potato starch aerogel).

Polysaccharide aerogels have recently been used extensively for enhancing the bioavailability of poorly watersoluble drugs. The release tests of crystalline ibuprofen and loaded tablet-shaped potato starch aerogel were carried out in both simulated gastric fluid (pH 1.2) and intestinal fluid (pH 7.4). Figure 8a shows the release kinetics of crystalline ibuprofen within SGF and SIF. In the first few minutes, crystalline ibuprofen was rapidly dissolved either partially (in case of SGF) or totally (in case of SIF). Enhanced dissolution of ibuprofen at basic pH is related to the common behavior of acidic drugs. When ibuprofen is dissolved in dissolution medium, some –COOH acidic functional groups of ibuprofen are ionized into –COO^-^ and H_3_O^+^ according to the following equilibrium:

(4)R--COOH+H2OR--COO-+H3O+

**Figure 9:**
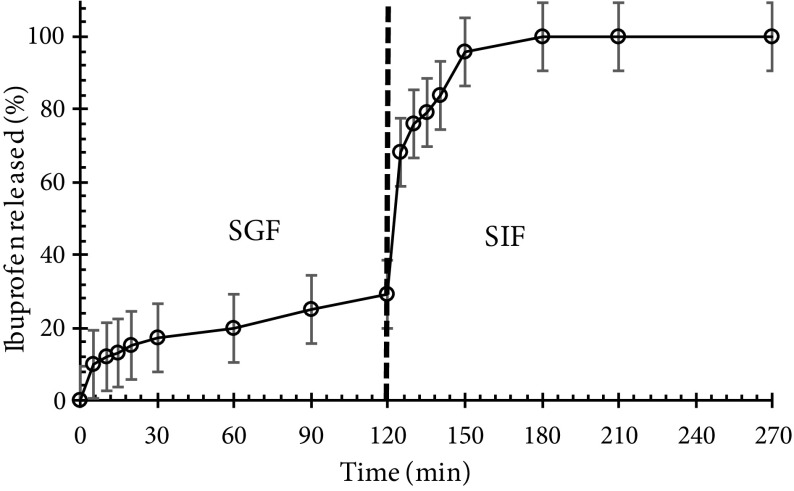
Ibuprofen release in SGF (30 min) and SIF (120 min).

If ibuprofen is dissolved in basic medium, some H_3_O^+^ ions react with OH^-^ groups of basic medium and the concentration of H_3_O^+^ ions decreases. Therefore, the equilibrium shifts right, which is in favor of ibuprofen dissolution. But when ibuprofen is dissolved in acidic medium, the concentration of H_3_O^+^ ions increases, and the equilibrium shifts left. Hence, the solubility of ibuprofen in SIF is greater than in SGF. This is a common behavior expected for acidic drugs. In the research performed by Veres et al., solubility values of ibuprofen, ketoprofen, and triflusal were measured in the order of 0.06, 0.26, and 0.69 mg/mL, respectively at pH 2 and 37 °C (acidic medium), while at pH 6.7 and 37 °C (basic medium), the solubility of the 3 compounds was measured approximately at 0.7 mg/mL [35]. Xu et al. showed that the release rate of ibuprofen in SGF was slower than in SIF. It took 80 h to release 10 wt% of ibuprofen from mesoporous silica spheres in SGF, but it only took 70 h to release 100 wt% of ibuprofen in SIF [37]. It was found that the release of acidic drugs such as ibuprofen was very slow in acidic SGF. Figure 8b shows the ibuprofen release profile from tablet-shaped potato starch aerogel in SGF and SIF. This Figure shows the semiretarded release profile for ibuprofen at pH 1.2. During the first 20 min, about ca. 20% of the total loaded ibuprofen was released by a slow delivery step, ca. 55% of the drug was released after 5 h of the dissolution test. At pH 7.4, during the first 90 min, a fast dissolution rate occurred. Hereafter, a sustained release up to reaching a plateau was recorded. During these 90 min, tablet-shaped potato starch aerogel released ca. 79% of the total loaded ibuprofen. It is evident that the dissolution rate of ibuprofen from potato starch aerogel matrix at pH 7.4 (basic medium) was faster than at pH 1.2 (acidic medium). This release behavior was related to the specific interaction established between the matrix and ibuprofen as a function of pH, but not to the solubility of ibuprofen. The results of the release of ibuprofen as a class II drug meet with the results of celecoxib (class II drug) release obtained by Khazraei and coworkers. They showed that the release rate of celecoxib in SGF was slower than in SIF. It took 120 min to release approximately 80% of celecoxib from alumina nanostructures in SGF, but it only took 10 min to release the same amount of celecoxib in SIF [38]. In drug release from a biodegradable and water-soluble matrix, diffusion, dissolution, and matrix erosion phenomena are implicated. Due to the high hydrophilicity of potato starch aerogels, their porous network leads to rapid collapse in an aqueous medium. Hence, drug molecules loaded into the matrix can be released to the buffer solution. The release kinetics determined by Mehling et al. at pH 7.2 show delivery of ca. 80% of ibuprofen from silica aerogel and alginate aerogel after 30 and 90 min, respectively [14]. For the ibuprofen-potato starch aerogel tablet studied in this work, about ca. 80% of total loaded ibuprofen was released after 90 min in pH 7.4. Figure 9 shows the release behavior of the loaded ibuprofen in two simulated mediums: gastric medium (pH 1.2) for 120 min and then intestinal medium (pH 7.4) for 60 min. This experiment was performed in order to mimic the behavior of the tablets after oral administration. It was found that potato starch aerogel matrix released ca. 29% of the total loaded ibuprofen in the gastric medium during the first 120 min. Then, the replacement of the gastric media with intestinal media resulted in the higher dissolution of ibuprofen. At pH 7.4, the release reached a plateau after 60 min at 100% release. This suggested that ibuprofen was encapsulated in the synthesized matrix. Figure 9 shows an approximately 10% increase in the dissolution of ibuprofen. Therefore, it could be concluded that by immersing the aerogel matrix, which was already swollen inside the gastric media, with the intestinal solution, the swelling and erosion of the matrix accelerated and ibuprofen release increased [33]. The release mechanisms of the drug from this matrix include the entrance of the dissolution medium into the matrix, matrix swelling, dissolution of the drug in the medium, diffusion of the drug through the gel layer, and erosion of the swollen matrix [39]. One of these phenomena or a combination of them control the release mechanism. During release kinetics at pH 7.4, the release of the ibuprofen molecules from the potato starch aerogel matrix occurred faster (60 min) than the degradation of the aerogel matrix by erosion. Therefore, the release of ibuprofen from potato starch aerogel matrix was predominately due to a diffusion process as well as swelling and erosion of the matrix. To better understand the release mechanism, the release data of ibuprofen in SIF were fitted by kinetic models of Korsmeyer–Peppas (Eq. 5), and Higuchi (Eq. 6). These mathematical models are presented by the following equations:

(5)qt=Kt

(6)qtq∞=Ktn

**Figure 10:**
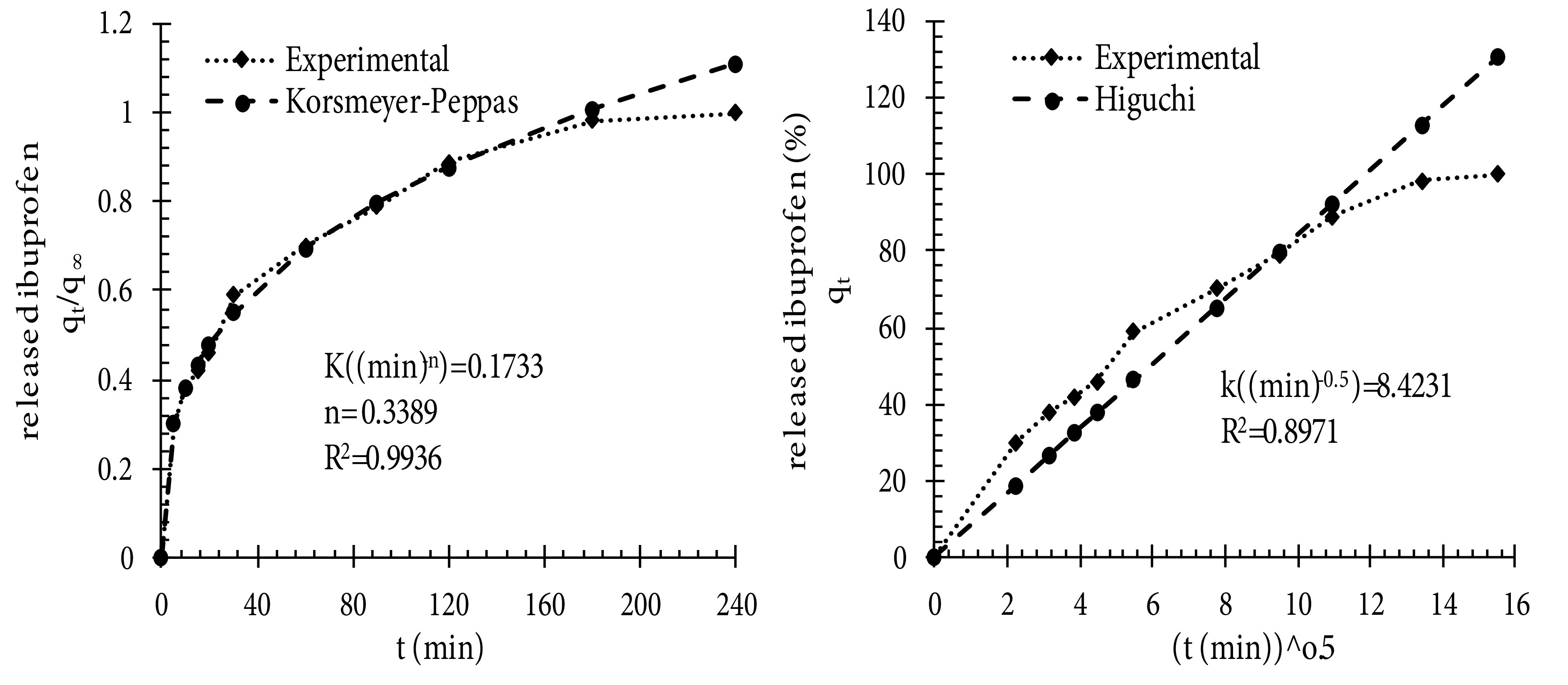
Experimental data of ibuprofen release from potato starch aerogel in SIF and the corresponding values predicted by the fitted model.

Where q_t_ is the amount of drug released at time t; q_∞_ is the amount of drug released at infinite time; n is the diffusion constant indicating the release mechanism, and K is the kinetic constant [39]. Figure 10 shows the curve fitting of the experimental data with the kinetics models of Korsmeyer–Peppas and Higuchi in simulated intestinal fluid. Regarding Figure 10, the best agreement was obtained with the Korsmeyer–Peppas model (R^2^ = 0.9936). Since the Korsmeyer–Peppas model takes into account the processes of swelling and erosion of the matrix [33], and the Higuchi model considers the Fickian diffusion [39], therefore, the release of ibuprofen from this matrix depends on diffusion and erosion. It could be concluded from the in vitro experiments results that loading poorly water-soluble drugs into potato starch aerogel matrix is the promising method for increasing their dissolution and bioavailability.

## 4. Conclusion

This work employed the design of experiment software for the first time to determine the effective parameters in the synthesis of potato starch aerogels and examine the effect of these parameters on the desired response (specific surface area). P-value of the model (0.000 <0.05 [significance level]) and lack-of-fit (0.097 >0.05 [significance level]) showed that the model used to optimize the experiments was sufficiently valid. The statistical analysis results showed that T_R_= 4◦, x, and t_S.E_ directly affected the response while the square of the main effect (x*x and t_S.E_ ∗ t_S.E_) had a reverse impact on the specific surface area. Surface plots showed that to synthesize aerogels with proper specific surface area, x, t_S.E_, and t_R_ should be in the range of 7.5%–15%, 16–25 h, and 3–5.5 days, respectively. Response optimization predicted optimal surface area of 65.6 m^2^/g in optimal values of 24 h, 4.7 days, and 14 wt% for the time period of solvent exchange, retrogradation time, and wt% starch in sol, respectively, at retrogradation temperature of 4◦. Ibuprofen was selected as a model drug and loaded into the optimized tablet-shaped potato starch aerogel by adsorption from an ibuprofen/ethanol solution during the last step of the solvent exchange process in order to improve its bioavailability. Adsorption isotherm showed the maximum adsorption capacity and specific loading of 290 mg/g and 4.5 ×10^-3^ g/m^2^, respectively. The results indicated that the Freundlich equation provided a good mathematical model to describe the equilibrium adsorption. Release tests showed that by loading ibuprofen into potato starch aerogel in molecular form, the release rate increased in both SGF and SIF. An enhanced dissolution of ibuprofen at pH 7.4 is due to the common behavior of acidic drugs. In order to simulate the behavior of the aerogels after oral administration, the gastric medium was replaced by an intestinal medium after 120 min. It was found that by immersing the swollen aerogel matrix within the intestinal solution, the swelling and erosion of the matrix accelerated, and ibuprofen release increased. Fitting the release data in a simulated intestinal fluid to the Korsmeyer–Peppas and Higuchi models, the best agreement was obtained with the Korsmeyer–Peppas model. It can be concluded that potato starch aerogels are promising drug delivery systems for controlling the release and enhancing the bioavailability of class II drugs in the BCS system.
